# Aspect Ratio Analysis for Ground States of Bosons in Anisotropic Traps

**DOI:** 10.6028/jres.101.056

**Published:** 1996

**Authors:** Kirill N. Ilinski, Alexander Moroz

**Affiliations:** School of Physics and Space Research, The University of Birmingham, Birmingham B15 2TT, United Kingdom; Institute of Spectroscopy, Russian Academy of Sciences, Troitsk, Moscow Region 142092, Russia; Institute of Physics, Na Slovance, CZ-180 40 Praha 8, Czech Republic; School of Physics and Space Research, University of Birmingham, Birmingham B15 2TT, United Kingdom

**Keywords:** analytic Ansatz, aspect ratio, Bose-Einstein condensation, ground state wavefunction

## Abstract

Characteristics of the initial condensate in the recent experiment on Bose-Einstein condensation (BEC) of ^87^Rb atoms in an anisotropic magnetic trap are discussed. Given the aspect ratio *R*, the quality of BEC is estimated. A simple analytical ansatz for the initial condensate wave function is proposed as a function of the aspect ratio which, in contrast to the Baym-Pethick trial wave function, can be used for any interaction strength, reproduces both the weak and the strong interaction limits, and which is in better agreement with numerical results than the latter.

## 1. Introduction

Bose-Einstein condensation (BEC) is a phenomenon where a macroscopic number of particles is in the ground state of the system at finite temperature. The phenomenon of BEC plays a significant role in many branches of physics [1]. Because of the presence of strong interactions, BEC has been inferred rather than directly observed so far. Recently, however, three different groups have reported the direct evidence of BEC in weakly interacting systems of atoms such as rubidium [2], lithium [3], and sodium [4], confined in anisotropic magnetic traps and cooled down to very low temperatures. These experiments show promise of becoming a new laboratory for quantum statistical phenomena that are inaccessible to other conventional techniques and of enabling us to experimental study phenomena that have been addressed only theoretically, such as spontaneous symmetry breaking and decay of unstable macroscopic states. They may also advance our understanding of superconductivity and superfluidity in more complex systems. Moreover, the technology used in the experiments has the possibility to be extended to produce a veritable atomic laser that is bound to have many applications in pure science and technology [5].

In recent experiments in the system of rubidium atoms [2] and sodium atoms [4], the onset of BEC is signalled by a narrow peak on top of a broad thermal velocity distribution centered at zero velocity. This peak exhibits the nonthermal, anisotropic velocity distribution expected of the minimum-energy quantum state of the magnetic trap in contrast to the thermal, isotropic velocity distribution observed in the broad uncondensed fraction. The parameter which characterizes the asymmetry of the velocity distribution function is the so called *aspect ratio* $R\equiv \sqrt{\langle p_{z}^{2}\rangle /\langle p_{x}^{2}\rangle }$. In the experiment of Anderson et al. [2], rubidium atoms are initially trapped and cooled down in a strong magnetic trap which can be described as a three-dimensional (3*D*) harmonic potential cylindrically symmetric about the *z*-axis, with tunable frequency *ω_z_* (in the *z* direction) and *ω*_⊥_ = *ω_z_*/*λ* (in the *xy*-plane), with the asymmetry parameter $\lambda =\sqrt{8}$. The corresponding oscillator lengths are *a*_⊥(_*_z_*_)_ = (*ℏ*/*mω*_⊥(_*_z_*_)_)^1/2^ = 1.25(0.74)×10^−4^ cm, where *m* is the atomic mass. After some time, the cloud of rubidium atoms is adiabatically released to a weaker magnetic trap whose spring constants are 10 times weaker than when BEC forms. The condensate is then examined after ballistic expansion from the weak trap. The ballistic expansion is properly modelled numerically by a self-consistent wavefunction calculated by Holland and Cooper [6] which generalizes previous investigation of the symmetric evolution [7].

Similarly to previous recent studies [8-10], we shall confine ourselves to the system of ^87^Rb atoms and we will discuss the characteristics of the initial condensate formed. Although the characteristics of the initial condensate have not been measured directly yet, there is hope that it will be possible so in future experiments.

A theoretical picture of the initial condensate was produced within the Hartree-Fock (HF) approximation [8–10] using the Ginzburg-Pitaevskii-Gross (GPG) energy functional [11] and associated with it the Nonlinear Schrödinger Equation (NSE). Excitations, using the technique of the Bogoliubov transformation, have been described by Fetter [12]. Baym and Pethick [8] have gained an insight into the problem by assuming that, similarly to the noninteracting case, a gaussian gives a reasonable variational ground-state wave function, the only effect of interactions being a renormalization of oscillator frequencies. They show that the first effect of interactions is to reduce the density of the cloud of particles in the central region from the free particle situation and expand it in the transverse direction. These qualitative features were confirmed by Edwards et al. [9] and Dalfovo and Stringari [10] by solving the NSE numerically.

**Fig. 1:**
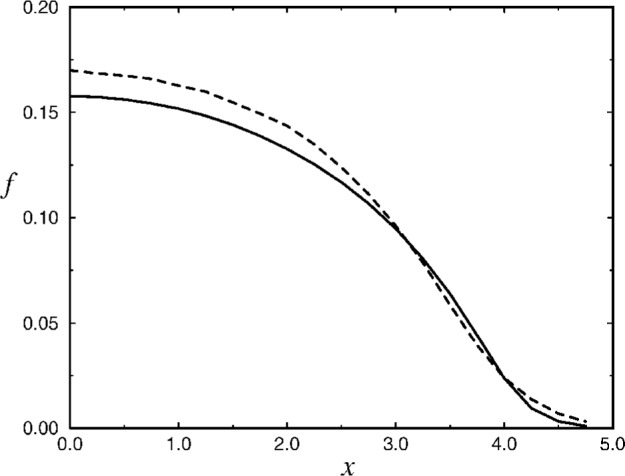
A comparison of the *x*-dependence of the numerical solution of NSE for the ground state [10] (dashed line) and our approximate solution (solid line) in the case of *N* = 5000 atoms of ^87^Rb, when *A* = 502 and *C*_ansatz_ = 2.2. The value of the aspect ratio, *R* (*A*, *λ*) = 2.3, is taken from Ref. [10].

In the present paper it is shown that although, qualitatively, the Baym and Pethick (BP) scenario [8] is correct, nevertheless, the BP wave function does not describe BEC regime well. As we show in the next section, this can be explained by the fact that the BP wave function is actually the square root of the first order density function in the high temperature expansion of the partition function of the *δ* -function interacting Bose gas in the kinetic energy, and hence describes high temperature properties of the system. Higher order corrections are needed to obtain low temperature properties such as BEC in agreement with the numerics. In particular, this explains the unreasonably high aspect ratio in BP estimations (up to 4.2, i.e., 2.5 higher than in the noninteracting case). All these prompt a search for another trial variational function for the ground state. In Sec. 3 we derive a simple analytical Ansatz (see Eq. (7) and Fig. 1) which, for a given numerical value of the aspect ratio, describes well the ground state properties of the system for all values of the interaction strength. The Ansatz interpolates smoothly between the weak and the strong interaction case. It is worthwhile to notice that the latter case cannot be described by the BP wave function and, instead, it is described by the Thomas-Fermi approximation in Ref. [8]. Using this Ansatz, correlation effects can be considered [13].

## 2. Baym-Pethick Trial Wave Function and High Temperature Expansion

Baym and Pethick argued [8] that the initial condensate wave function in the region of weak and up to intermediate interactions can be well approximated by a Gaussian. Let us take a different point of view and ask under which condition this Gaussian-like profile of the initial condensate wave function can be actually derived.

If interactions between atoms are neglected, the physical system is equivalent to the system of noninteracting harmonic oscillators. In the latter case, one can show that the aspect ratio in thermal equilibrium is a monotonically increasing function of the inverse temperature *β* = 1/*T*,

$$R\left(\lambda ,\beta \right)=\sqrt{\lambda \frac{1/2+{\left(e^{\beta \lambda \omega_{⊥}}-1\right)}^{-1}}{1/2+{\left(e^{\beta \lambda \omega_{⊥}}-1\right)}^{-1}}}.$$

At high temperatures *R* ≈ 1, and for low temperatures $R\approx \sqrt{\lambda }$. In the latter case, the dominant contribution to the aspect ratio is given by the ground state of the system and reflects its anisotropy.

In the presence of the interactions, the Hamiltonian of the system can be written as

$$H=\frac{\hbar^{2}}{2m}\int dV\left[\nabla \psi^{+}\nabla \psi +a_{⊥}^{-4}\left(\rho^{2}+\lambda^{2}Z^{2}\right)\psi^{+}\psi +4\pi l\psi^{+}\psi^{+}\psi \psi \right],$$ (1)

where *ρ*^2^ = *x*^2^ + *y*^2^, *a*_⊥_ and *a_z_* are oscillator lengths, and *l* is the *s*-wave scattering length [8,14]. Qualitatively, the temperature dependence of *R*(*λ*, *b*) preserves the main features of the noninteracting case. At high temperatures, the interaction is irrelevant and *R* ≈ 1. At low temperatures (for sufficiently small fraction of atoms out of BEC), the HF approximation is justified and the ground state wave function (giving the main contribution to the aspect ratio) satisfies the NSE. After rescaling of variables [10], the NSE can be written as

$$\left[-\Delta +x^{2}+y^{2}+\lambda^{2}z^{2}+A{\left|\psi \left(r\right)\right|}^{2}\right]\psi \left(r\right)=2C\psi \left(r\right).$$ (2)

Here, *A* = 8π*lN*/*a*_⊥_ characterizes the interaction strength, *N* is the number of particles in the condensate (*A* ~ 520 for *N* ~ 5000 [15]), and *C* = *μ*/(*ℏω*_⊥_) > 0, *μ* being the chemical potential. In the case of large condensate fraction (strongly interacting case, *A* ≫ 1), the kinetic term can be neglected [8,10] and the ground state (normalized to unity) wave function is given by the Thomas-Fermi approximation,

$$f^{2}\left(r\right)=\frac{1}{A}\left(2C-x^{2}-y^{2}-\lambda^{2}z^{2}\right)\Theta \left(2C-x^{2}-y^{2}-\lambda^{2}z^{2}\right),$$ (3)

where 2*C* = [15*λA*/(8π)]^2/5^, and *Θ* is the Heaviside step function. The aspect ratio, *R*(*A*, *λ*), is increasing function of *A*, and the ground state solution Eq. (3) takes on its maximal possible value (*R* = *λ*) among all ground state solutions to the NSE. In the present case $\left(\lambda =\sqrt{8}\right)$, this means that the maximal effect of interactions is to raise the value of the aspect ratio on 67 % with respect to the noninteracting case. Moreover, as shown by Dalfovo and Stringari [10], the aspect ratio for *A* = 520 (corresponding to *N* = 5000 atoms in BEC) is *R* = 2.3, i.e., 37 % higher than in the noninteracting case.

Let us now return to the BP wave function [8]. In order to simplify the derivation of the BP wave function from the high-temperature expansion, let us for a while, such as in Ref. [8], neglect the anisotropy of the oscillator potential [16]. Because the kinetic energy of particles in the system is approximately 200 times smaller than the characteristic interaction energy [8], it is reasonable to consider the expansion of the partition function of the system, *Z* (*β*, *μ*), in powers of the kinetic term,

$$Z\left(\beta ,\mu \right)=\int D\psi^{+}\left(x,\tau \right)D\psi \left(x,\tau \right)e^{S_{0}}\sum_{n=0}^{\infty }\frac{{\left[-\hbar^{2}/\left(2m\right)\right]}^{n}}{n!}{\left(\int_{0}^{\beta }\int dV\nabla \psi^{+}\nabla \psi \right)}^{n}\equiv \sum_{n=0}^{\infty }Z_{n}\left(\beta ,\mu \right).$$ (4)

Here *S*_0_ is the “unperturbed” action,

$$S_{0}=\int_{0}^{\beta }\int dV\left\{\frac{\partial \psi^{+}}{\partial \tau }\psi -\frac{\hbar^{2}}{2m}\left[\left(a_{⊥}^{-4}r^{2}-\frac{2m}{\hbar^{2}}\mu \right)\psi^{+}\psi +4\pi l\psi^{+}\psi^{+}\psi \psi \right]\right\},$$

with fields satisfying the periodic boundary conditions, *ψ*^+^(*x*, *β*) = *ψ*^+^(*x*, 0) and *ψ*(*x*, *β*) = *ψ*(*x*, 0). Expansion [Eq. (4)] is the high temperature expansion and we are not exactly in the BEC regime. However, the HF approximation is avoided.

In the first term (corresponding to *n* = 0) of the sum in Eq. (4) one finds a product of partition functions of the anharmonic oscillators {*H_x_*}, labelled by the space point *x*. Using the lattice approximation *x* = *l* (*m*_1_, *m*_2_, *m*_3_) ≡ *lm* with the scattering length *l* (the smallest length in the system) being the lattice spacing, one has for field operators $\psi^{+}\left(x\right)=\psi_{m}^{+}/\sqrt{l^{3}}$ and $\psi \left(x\right)=\psi_{m}/\sqrt{l^{3}}$, and

$$Z_{0}\left(\beta ,\mu \right)=\prod_{m}Z^{m}\left(\beta ,\mu \right).$$

Here, *Z^m^*(*β*, *μ*) is the partition function of the one-site Hamiltonian *H_m_*,

$$H_{m}=\frac{\hbar^{2}}{2ml^{2}}\left[\frac{l^{4}}{a_{⊥}^{4}}\left(m_{1}^{2}+m_{2}^{2}+m_{3}^{2}\right)-\frac{2ml^{2}}{\hbar^{2}}\mu -4\pi \right]\psi^{+}\left(x\right)\psi \left(x\right)+4\pi {\left(\psi^{+}\left(x\right)\psi \left(x\right)\right)}^{2}.$$

After redefining parameters,

$$\begin{array}{ll} \mu \to \tilde{\mu }\equiv \frac{2ml^{2}}{\hbar^{2}}\mu , & \beta \to \tilde{\beta }\equiv \frac{\hbar^{2}}{2ml^{2}}\beta ,\\ b_{m}=\frac{l^{4}}{a_{⊥}^{4}}\left(m_{1}^{2}+m_{2}^{2}+m_{3}^{2}\right) & \end{array}$$

one has

$$Z^{m}\left(\beta ,\mu \right)=\sum_{k=0}^{\infty }\exp\left(-4\pi \tilde{\beta }k^{2}+\tilde{\beta }\tilde{\mu }k+4\pi \tilde{\beta }k-\tilde{\beta }kb_{m}\right).$$

Let us assume that the value of the chemical potential $\tilde{\beta }\tilde{\mu }$ is negative and order of 10 (self-consistency of this assumption will be shown below). By substituting *T* ≃ 10^−7^ K for the temperature, one has $4\pi \tilde{\beta }≃12\pi \times 10^{3}$, and

$$\begin{array}{c} Z^{m}\left(\beta ,\mu \right)=1+e^{\tilde{\beta }\tilde{\mu }-\tilde{\beta }b_{m}}\left(1+O\left(e^{-2.5\pi 10^{4}}\right)\right)\\ ≃1+e^{\tilde{\beta }\tilde{\mu }-\tilde{\beta }b_{m}}. \end{array}$$ (5)

The resulting partition function

$$Z_{0}\left(\beta ,\mu \right)=\prod_{m}\left(1+e^{\tilde{\beta }\tilde{\mu }-\tilde{\beta }b_{m}}\right),$$

leads to the distribution function 〈*ψ*^+^(*x*)*ψ*(*x*)〉 of the Fermi-Dirac type,

$$\langle \psi^{+}\left(x\right)\psi \left(x\right)\rangle =\frac{1}{1+e^{-\tilde{\beta }\tilde{\mu }+\tilde{\beta }b_{m}}},$$

with the chemical potential $\tilde{\mu }$ to be determined from the normalization condition,

$$N=\int \frac{dV}{1+e^{-\tilde{\beta }\tilde{\mu }+\tilde{\beta }b_{m}}}.$$ (6)

Let *N* ≃ 5000 be the number of particles in the system. Using $l^{4}/a_{⊥}^{4}≃2\times 10^{-10}$, and, neglecting 1 in the denominator of Eq. (6), one gets $e^{\tilde{\beta }\tilde{\mu }}≃1.3\times 10^{-10}N/\pi^{3/2}$ which implies $\tilde{\beta }\tilde{\mu }≃\ln\left(10^{-7}\right)≃-16.1$, in full accord with our assumption.

We cannot justify our treatment for low temperatures. Nevertheless, under the assumption that our expansion holds up to low temperatures, the profile of the square root of the density function *ψ*_0_(*x*) of the system turns out to be the BP trial wave function [8],

$$\psi_{0}\left(x\right)=\sqrt{\langle \psi^{+}\left(x\right)\psi \left(x\right)\rangle }=\mathrm{const}\times \exp\left(-\frac{r^{2}}{2{\tilde{a}}_{⊥}^{2}}\right),$$

where ${\tilde{a}}_{⊥}=a_{⊥}^{2}/\left(1\sqrt{\tilde{\beta }}\right)$ The very fact that the BP wave function is the first order result suggest that it may not describe BEC well. Therefore, the aspect ratio obtained from the BP wave function may not be reliable. Moreover, the profile of the BP function differs significantly from the exact ground state wave function calculated numerically in Refs. [9,10].

## 3. New Analytical Ansatz

We shall show that the ground-state wave function *f* (***r***) of the system can be well described by a simple analytical Ansatz (cf. Fig. 1),

$$f^{2}=\frac{1}{A}W\left[A\;\exp\left(4C-\rho^{2}-R^{2}\left(A,\lambda \right)z^{2}\right)\right],$$ (7)

where *W*(*x*), defined as the principal branch (regular at the origin) solution to the Eq. *W*e*^W^* = *x* [17], is a standard MAPLE function [18]. The constant *C* in Eq. (7) is to be determined from the normalization condition, ∫ *f* ^2^(*r*)d^3^*x* = 1. To approximate the ground-state solution to the NSE, the value of *R* should be supplied from the numerical solution [10]. On the other hand, given the experimental value of *R*, our Ansatz can serve to reproduce the profile of the ground state.

Equation (2) is a nonlinear equation and, in the present case, no exact solutions are known except for the two limiting cases, *A* = 0 and Δ*f* ≪ *Af*^3^. In what follows, we shall construct our Ansatz to reproduce correctly the two limiting cases and to interpolate smoothly between them as the interaction changes. Note that in the strong interaction limit the Baym-Pethick trial wave-function cannot be used at all and the Thomas-Fermi approximation was used in Ref. [8] in this limit. The main point in our derivation is to use instead of the second order differential equation [Eq. (2)] a set of *first* order differential equations which reproduce correctly both the noninteracting limit (*A* = 0) and the strongly interacting limit (Δ*f* ≪ *Af*^3^). In this sense, our Ansatz will be exact in the first derivatives. In the noninteracting limit, Eq. (2) reduces to the stationary Schrödinger equation for an anisotropic oscillator and the (normalized to unity) ground state wave function is

$$f\left(r\right)=\frac{\lambda^{1/4}}{\pi^{-3/4}}\;\exp\;\left[-\frac{1}{2}\left(x^{2}+y^{2}+\lambda z^{2}\right)\right],$$ (8)

with *C* = 2 + *λ* and, in agreement with our previous discussion, $R=\sqrt{\lambda }$. One notice that *f* (***r***) satisfies the set of first order differential equations,

$$\partial_{1}f=-xf,\;\partial_{2}f=-yf,\;\lambda^{-1}\partial_{3}f=R^{-2}\left(\lambda \right)\partial_{3}f=-zf.$$ (9)

On the other hand, if Δ*f* ≪ *Af*^3^, *Af*^3^/*λ*, the (normalized to unity) ground-state wave function is given by the Thomas-Fermi approximation [see Eq. (3)], and

$$\begin{array}{l} Af^{2}\partial_{1}f\approx -xf,\;Af^{2}\partial_{2}f\approx -yf,\\ \lambda^{-2}Af^{2}\partial_{3}f=R^{-2}\left(\lambda \right)Af^{2}\partial_{3}f\approx -zf. \end{array}$$ (10)

Now, the first order differential equations (8,10) can be combined into the variational principle, *E* [*f*] = ∫ *Σ_j_ P_j_ P_j_*d^3^***r***, where *P*_1_= [(1+*Af*^2^)∂_1_+*x*]*f, P*_2_=[(1+*Af*^2^*)*∂_2_+*y*]*f, P*_3_ [*R*^−2^ (*A*, *λ*)(1+*Af*^2^*)*∂_3_+*z*]*f.* The actual form of *P*_3_ in the asymmetric case is fixed by the requirement to make simultaneous integration of the first order differential equations [Eq. (11)] possible. The variational principle implies the following set of first order equations for the ground state wave function,

$$\partial_{\rho }\;f=-\frac{\rho f}{1+Af^{2}},\;\partial_{3}\;f=-R^{2}\left(A,\lambda \right)\frac{zf}{1+Af^{2}}$$ (11)

**Fig. 2:**
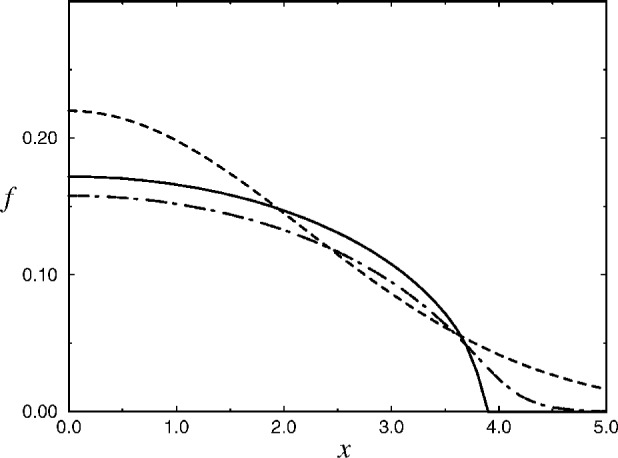
A comparison of the *x*-dependence of the ground-state solution of Baym and Pethick (solid line), strong limit (dot-dashed line) and our approximate solution (dashed line). The same values of the parameters were used as for Fig. 1.

**Fig. 3:**
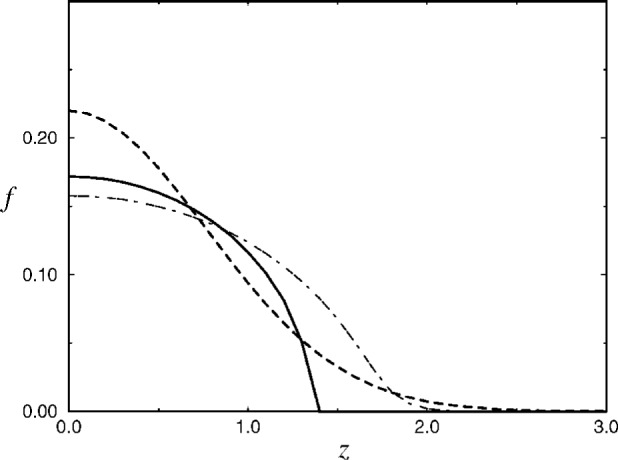
*z*-dependence of the ground-state solution of Baym and Pethick (dot-dashed line), strong limit (solid line) and our approximate solution (dashed line).

By integrating Eqs. (11) one obtains

$$fe^{Af^{2}/2}=\exp\left[2C-\rho^{2}/2-R^{2}\left(A,\lambda \right)z^{2}/2\right],$$

from which our Ansatz [Eq. (7)] follows immediately. Provided that *ρ*^2^ + *R* ^2^(*A*, *λ*)*z*^2^ ⩽ 4*C*, *f* can be found explicitly using successive iterations,

$$f^{2}=\frac{1}{A}\ln\frac{s^{2}}{f^{2}}=\frac{1}{A}\ln\frac{s^{2}}{\frac{1}{A}\ln\frac{s^{2}}{\frac{1}{A}\ln\frac{s^{2}}{\ldots }}}>0,$$ (12)

where *s* = exp [2*C* − *ρ*^2^/2 − *R*^2^(*A*, *λ*) *z*^2^]. Obviously, *f* (***r***) given by Eq. (7) reproduces correctly the ground state wave function both in the noninteracting limit (*A* = 0) and in the strongly interacting limit (*Af*^2^ ≫ 1), and interpolates smoothly between the two limiting cases in the intermediate region (see Figs. 2,3). One can verify that if (*Af*^2^ ≫ 1) then Δ*f*~ − [1/(*Af*^2^)^2^](*x*^2^ + *y*^2^ + *R*^2^(*A*, *λ*) *z*^2^ + 3*Af*^2^) *f*, and the kinetic term is suppressed by the factor (*Af*^2^)^−2^ with respect to the remaining terms in Eq. (2). Our Ansatz can substitute for the BP trial wave function and can play the role of a new trial wave function in various variational calculations. Given experimental values of the aspect ratio, our Ansatz can be effectively applied to describe the initial BEC wave function and to calculate all relevant properties of the initial BEC. We believe that the derivation of our Ansatz can be extended to deal with excited states too.

## 4. Discussion and Conclusions

In this paper, only the properties of the initial condensate were considered. To find the connection with experiment, it is necessary to discuss properties of the system during the transition from the strong trap to the weak trap and its subsequent ballistic expansion from the weak trap. Obviously, the characteristics of the system will change after the expansion and will strongly depend on the condition of the expansion (i.e., whether it is adiabatic or abrupt). Nevertheless, the present study allows us to give an upper bound for the aspect ratio of the condensate after its expansion directly from the strong trap, i.e., in the absence of the intermediate weak trap, as it took place in [2]: *the aspect ratio of the final system is always lower than that calculated for the initial condensate*. Indeed, after the expansion, (i) there is no more anisotropic potential applied, (ii) the self-interaction of bosons, which gives rise to the increase of the aspect ratio with respect to the noninteracting case, is decreasing. Note that if one can measure the aspect ratio directly after the expansion from the strong trap as it should be done in future experiments, it would be possible to estimate the real number of the particles in the condensate.

We want to emphasize that, as can be found from comparison of the results obtained from the BP wave function, the Thomas-Fermi approximation, and from the exact numerical solution, the aspect ratio may be a very sensitive characteristic of the wave function and may change considerably even when other characteristics are not changed by a perturbation (such as an external potential or an interaction). That is why we expect that taking into account correlation effects may lead to considerable changes in the aspect ratio, although for other characteristics the Hartree-Fock approximation will give correct results. We will consider this question in detail in a forthcoming paper [13].

Summarizing, given the value of the aspect ratio, both the profile of the ground state and the quality of BEC can be estimated. This allows one to estimate the number of particles in the initial condensate. We showed that the Baym-Pethick trial wave-function (i) is only the first order approximation in the high temperature expansion for the system and (ii) does not describe the condensate wave function accurately even for weak and intermediate interactions. Note that in the strong interaction limit the Baym-Pethick trial wave-function cannot be used at all and instead the Thomas-Fermi approximation was used in Ref. [8]. In order to describe the ground state of the initial condensate in the whole range of the interparticle interactions, we proposed a simple analytical Ansatz which, in contrast to the BP trial wave function, reproduces correctly both the weak and the strong interaction limit and interpolates smoothly between the two limiting cases as interaction changes.

We want to thank J. M. F. Gunn for raising our interest in the problem and for many encouraging discussions. We are also grateful to K. Burnett, F. Dalfovo, M. Holland, and C. Pethick for discussion, and P. Cooper for careful reading of the manuscript. This work was partially supported (K. I.) by the Grant of Russian Fund of Fundamental Investigations N 95-01-00548, the UK EPSRC Grant GR/J35221, and (A. M.) by the UK EPSRC Grant GR/J35214, and by the Grant Agency of the Czech Republic under Project No. 202/93/0689.
